# Genomic Epidemiology of SARS-CoV-2 From Mainland China With Newly Obtained Genomes From Henan Province

**DOI:** 10.3389/fmicb.2021.673855

**Published:** 2021-05-20

**Authors:** Ning Song, Guang-Lin Cui, Qing-Lei Zeng

**Affiliations:** ^1^Center for Reproductive Medicine, Henan Key Laboratory of Reproduction and Genetics, The First Affiliated Hospital of Zhengzhou University, Zhengzhou, China; ^2^Department of Clinical Laboratory, The First Affiliated Hospital of Zhengzhou University, Zhengzhou, China; ^3^Department of Infectious Diseases, The First Affiliated Hospital of Zhengzhou University, Zhengzhou, China

**Keywords:** SARS-CoV-2, whole-genome sequence, tMRCA, evolutionary rate, Henan Province, Mainland China

## Abstract

Even though the COVID-19 epidemic in China has been successfully put under control within a few months, it is still very important to infer the origin time and genetic diversity from the perspective of the whole genome sequence of its agent, SARS-CoV-2. Yet, the sequence of the entire virus genome from China in the current public database is very unevenly distributed with reference to time and place of collection. In particular, only one sequence was obtained in Henan province, adjacent to China's worst-case province, Hubei Province. Herein, we used high-throughput sequencing techniques to get 19 whole-genome sequences of SARS-CoV-2 from 18 severe patients admitted to the First Affiliated Hospital of Zhengzhou University, a provincial designated hospital for the treatment of severe COVID-19 cases in Henan province. The demographic, baseline, and clinical characteristics of these patients were described. To investigate the molecular epidemiology of SARS-CoV-2 of the current COVID-19 outbreak in China, 729 genome sequences (including 19 sequences from this study) sampled from Mainland China were analyzed with state-of-the-art comprehensive methods, including likelihood-mapping, split network, ML phylogenetic, and Bayesian time-scaled phylogenetic analyses. We estimated that the evolutionary rate and the time to the most recent common ancestor (TMRCA) of SARS-CoV-2 from Mainland China were 9.25 × 10^−4^ substitutions per site per year (95% BCI: 6.75 × 10^−4^ to 1.28 × 10^−3^) and October 1, 2019 (95% BCI: August 22, 2019 to November 6, 2019), respectively. Our results contribute to studying the molecular epidemiology and genetic diversity of SARS-CoV-2 over time in Mainland China.

## Introduction

Severe acute respiratory syndrome coronavirus 2 (SARS-CoV-2) causes the severe respiratory disease coronavirus disease 2019 (COVID-19), which was first reported in Wuhan city, Hubei Province, China in December 2019 (Wu et al., 2020a; Zhou et al., 2020; Zhu et al., 2020), subsequently turning into a pandemic with devastating effects. As of February 20, 2021, there have been 110 million confirmed infections and 2.4 million reported deaths worldwide (https://www.who.int/emergencies/diseases/novel-coronavirus-2019). So far, China has successfully managed to contain the epidemic. However, the number of newly confirmed cases has been rising rapidly outside of China, especially in the USA, India, Brazil, and Russia.

Henan Province, which borders with Hubei Province to the north, has the third-largest population in China (94 m people). It contains many large cities, including Zhengzhou (10 million people), Nanyang (10 million people), Zhoukou (8 million people), Shangqiu (7 million people), and Zhumadian (7 million people). Henan Province has the most transportation links with Hubei Province. The shortest travel time by high-speed railway from Zhengzhou (the capital city of Henan Province) to Wuhan (the capital city of Hubei Province) is <2 h. Moreover, most people from the southern regions of Henan Province choose to work in Wuhan. By December 30, 2020, Henan Province had reported 1,273 local confirmed cases of COVID-19, including 22 deaths. Almost all of the cases in Henan Province were reported in January and February 2020, as described by our previous studies (Zeng et al., 2020a,b).

Tracking the on-going evolution and transmission patterns of SARS-CoV-2 from the perspective of genomic epidemiology may further our understanding of the COVID-19 pandemic and could help improve public-health measures for containing the virus. The first genome sequence of SARS-CoV-2, which was isolated from a 41-year old man who worked at Wuhan Huanan Seafood Wholesale Market in the city of Wuhan, was posted on January 11, 2020 (Wu et al., 2020b). Previous studies on the genomic epidemiology of SARS-CoV-2 in China were based on individual province or limited amount of genome sequences (Lu et al., 2020; Nie et al., 2020; Geidelberg et al., 2021). However, with the increasing number of genome sequences available in the public database, it is imperative to perform such analysis with more sequences sampling from wider area in longer time span. Notably, there was only one whole-genome sequence reported from Henan deposited in the Global Initiative on Sharing All Influenza Data (GISAID) (http://gisaid.org/) (Elbe and Buckland-Merrett, 2017). In the present study, we generated 19 genome sequences of SARS-CoV-2 strains from 18 severe or critically ill patients in Henan Province using metagenomic sequencing. Together with other 710 genome sequences sampled from Mainland China with sampling dates between 24 December 2019 and 22 July 2020, we employed advanced comprehensive methods to investigate the genetic diversity, evolution, and transmission patterns of SARS-CoV-2 in China. The demographic, baseline, and clinical characteristics of these patients were also described. Our study may provide valuable information for investigating the impact of the early public-health intervention on SARS-CoV-2 transmission and evolution in China with high resolution than previous studies.

## Materials and Methods

### Patients

According to the unified arrangement of the Henan provincial government, a total of 47 confirmed severe or critically ill patients were transported to and treated at the First Affiliated Hospital of Zhengzhou University between January 22, 2020 and March 7, 2020. All of these patients were diagnosed as severe or critically ill COVID-19 cases prior to their admission to the First Affiliated Hospital of Zhengzhou University. The classification criteria for disease severity were strictly in accordance with the national COVID-19 Control Plan.

### Ethics Statement

This study was approved by the ethics committee of the First Affiliated Hospital of Zhengzhou University (ethical approval number: 2020-KY-116). Inform consent was waived as the samples used for the present study were collected after routine laboratory testing, and the study was considered less than minimal risk to subjects by the aforementioned committee.

### Sample Collection and the Nucleic Acid Test of SARS-CoV-2

Throat swabs were collected from patients who were confirmed and hospitalized with COVID-19 at the First Affiliated Hospital of Zhengzhou University during February 2020 and screened for the presence of nucleic acid of SARS-CoV-2 in the Biosafety Level II Laboratory of the hospital. Patient demographic information was also collected on admission. The collection, transport, and nucleic acid test of specimens were carried out in strict accordance with relevant national regulations. Throat swab samples were inactivated at 56°C for 30 min. Total nucleic acid extraction was performed using the nucleic acid extraction kit (Shanghai BioGerm Medical Technology Co., Ltd.) following manufacturer's instructions. SARS-CoV-2 nucleic acid detection was conducted by a qRT-PCR method using the SARS-CoV-2 nucleic acid test kit (Shanghai BioGerm Medical Technology Co., Ltd.). The determination criteria for positive results are strictly in accordance with the manufacturer's instructions. The remaining positive nucleic acid samples were immediately stored at −80°C for subsequent sequencing analysis.

### Next-Generation Sequencing of the Complete SARS-CoV-2 Genomes

Next-generation sequencing of the complete SARS-CoV-2 genomes was performed by the BGI Company (Shenzhen, China). Briefly, host DNA was removed from the positive samples using DNase I, and the concentration of RNA samples was measured by Qubit RNA HS Assay Kit (Thermo Fisher Scientific, Waltham, MA, USA). DNA-depleted and purified RNA was used to construct the double-stranded (ds) circular DNA library with MGIEasy RNA Library preparation reagent set (MGI, Shenzhen, China), as follows: (1) RNA was fragmented by incubating with fragmentation buffer at 87°C for 6 min; (2) ds cDNA was synthesized using random hexamers with fragmented RNA; (3) ds cDNA was subjected to end repair, adaptor ligation, and 18-cycle PCR amplification; (4) PCR products were unique dual indexed (UDI), before going through circularization and rolling circle replication (RCR) to generate DNA nanoball (DNB)-based libraries. Negative controls prepared from nuclease-free water and total RNA isolated from human Michigan Cancer Foundation-7 (MCF-7) breast cancer cells were included. DNB preps of clinical samples were sequenced on the ultra-high-throughput DNBSEQ-T1 platform (MGI, Shenzhen, China) with a paired-end 100 nt strategy, generating average of 100 Gb sequencing data for each sample.

### Mutation Calling and Clade Assignment Analysis

To identify differences between 19 genome sequences from Henan Province and reference sequences, and to find out which clades they are from and where on the SARS-CoV-2 tree they fall, we used the Nextclade web tool (beta v0.10.0, https://clades.nextstrain.org/) to perform mutation calling and clade assignment. Here, clades are groups of related sequences that share a common ancestor. Of note, the Nextclade does not do a real phylogenetic analysis. Instead, clade assignment sequence-by-sequence was performed based on signature mutations. The clade-defining mutations (shown on https://clades.nextstrain.org/) are chosen such that assignment based on genotype works in most cases.

### Collation of SARS-CoV-2 National Dataset

To investigate the full genetic characterization of the complete genomes of SARS-CoV-2 in Mainland China, 710 complete genomes of SARS-CoV-2 available from the GISAID (http://gisaid.org/) were downloaded (Supplementary Table 1) as of October 23, 2020, and aligned with 19 full-length genomes of SARS-CoV-2 determined in the present study from Henan Province. No statistical methods were used to predetermine the number of genomes in the present study as we downloaded all available genomes of human-obtained SARS-CoV-2 strains. Notably, genome sequences that were from environment or duplicate samples, and those without exact collection dates and sampling locations, and those contained >5% Ns after mapping to the Wuhan-Hu-1 reference (GenBank accession number: MN908947.3) were discarded. The dataset used in the present study was also not randomized. The final dataset (“dataset_729”) included 729 genomes of SARS-CoV-2 strains from 17 provinces with sampling dates from December 24, 2019 to July 22, 2020. Of the 19 genomes of SARS-CoV-2 strains collected in the present study from Zhengzhou City for household registration in Henan Province, one was from Luohe City, one was from Luoyang City, one was from Nanyang City, two were from Shangqiu City, four were from Xinyang City, one was from Xuchang City, four were from Zhengzhou City, two were from Zhoukou City, and three were from Zhumadian City. We first aligned the collected dataset (“dataset_729”) using MAFFT v7.222 (Katoh and Standley, 2013) under an automatic algorithm and then manually edited the alignment using BioEdit v7.2.5 (Hall, 1999).

### Recombination Screening and Maximum-Likelihood Analysis

As recombination is a relatively frequent evolutionary mechanism in coronaviruses (Graham and Baric, 2010), we assessed the recombination of our dataset (“dataset_729”) by the Phi-test approach using SplitsTree4 v4.16.2 (Huson and Bryant, 2006) and all available recombination detection methods using the Recombination Detection Program (RDP) v4.100 (Martin et al., 2015). The best-fit nucleotide substitution model for “dataset_729” was identified according to the Bayesian information criterion (BIC) method, Akaike Information Criterion (AIC) method, Corrected Akaike Information Criterion (AICc) method, and Decision Theory Selection (DT) method, with three (24 candidate models) substitution schemes in jModelTest v2.1.10 (Darriba et al., 2012). To evaluate the phylogenetic signals of “dataset_729,” we performed likelihood-mapping analysis (Schmidt and Von Haeseler, 2007) using TREE-PUZZLE v5.3 (Schmidt et al., 2002), with 10,000 randomly chosen quartets for the dataset. Split network analysis was performed for “dataset_729” using a general time-reversible (GTR) characters transformation with a gamma distribution to describe among-site variation in the rate of nucleotide substitution (Γ), and a proportion of invariable (I) sites (GTR + Γ + I), with the NeighborNet method, which can be loosely thought of as a “hybrid” between the neighbor-joining (NJ) and split decomposition methods, implemented in SplitsTree4 v4.16.2 (Huson and Bryant, 2006). We estimated the maximum-likelihood (ML) phylogenetic tree for the dataset using RAxML v8.2.12 under the GTR + Γ + I nucleotide substitution model, which was identified as the best fitting model for ML inference by jModelTest v2.1.10 (Darriba et al., 2012). Branch support was inferred using 1 000 bootstrap replicates (Felsenstein, 1985), and trees were midpoint rooted. ML phylogenetic tree was visualized using FigTree v1.4.4 (http://tree.bio.ed.ac.uk/software/figtree/), after which we regressed root-to-tip genetic divergence obtained from the ML phylogeny against sampling dates to investigate the temporal molecular evolutionary signals for the dataset using TempEst v1.5 (Rambaut et al., 2016).

### Molecular Clock Phylogenetics

The Bayesian molecular clock phylogenies of SARS-CoV-2 for “dataset_729” were estimated under GTR + Γ + I nucleotide substitution model, a parametric exponential growth tree prior, and a strict molecular clock, which assumed constant evolutionary rates throughout the phylogeny, through a Markov chain Monte Carlo (MCMC) (Yang and Rannala, 1997) framework implemented in BEAST v1.10.4 (Drummond et al., 2012). A non-informative continuous-time Markov chain (CTMC) reference prior (Ferreira and Suchard, 2008) was used for the molecular clock rate. Bayesian analyses were run using BEAGLE v2.1.2 (Suchard and Rambaut, 2009) for computational enhancement. Bayesian analysis was run for 500 million MCMC steps with sampling parameters and trees every 50 000 generations. The convergence of the MCMC chains was inspected using Tracer v1.7.1 (Rambaut et al., 2018). Maximum clade credibility (MCC) summary tree was generated using TreeAnnotator v1.10.4 (Drummond et al., 2012) after discarding the first 10% as burn-in. MCC summary tree was visualized using FigTree v1.4.4 (http://tree.bio.ed.ac.uk/software/~figtree/).

### Nucleotide Sequence Accession Numbers in GISAID

Nucleotide sequences of complete genome of SARS-Cov-2 identified in this study have been deposited in the GISAID under accession numbers EPI_ISL_1040031 to EPI_ISL_1040049.

## Results

### Sequencing Result Summary

We collected 28 samples of nucleic acids extracted from 27 COVID-19 patients hospitalized in the first affiliated hospital of Zhengzhou University, which were then sent to BGI Company for next-generation sequencing. The total number of reading pairs among the 28 samples was between 209,838,632–1,540,364,746 (sequencing volume 41G~308G), and the average sample had 743,557,625 read pairs (average sequencing volume is 148G), as shown in Supplementary Table 2. The number of SARS-CoV-2 sequences per million (NSPM) sequencing data was between 0.08 and 12,907, and the average NSPM value was 748. Eight samples had NSPM values <1.5, and only one sample had genome coverage >90%; the remaining 20 samples contained 18 genomes with coverage >90% (Supplementary Table 2). The sequencing depth was between 0.12X and 6148X, the average sequencing depth was 363X, and there were 19 samples with a depth of more than 4X that could assemble a genome with coverage >90% (Supplementary Table 2). In the present study, a total of 19 samples assembled a genome with coverage of more than 90%. Mutation sites with genome coverage over 90% and sequencing depth over 5X are shown in Supplementary Figure 1.

Detail information on the SNP in the 19 Henan sequences is shown in Table 1 and Supplementary Figure 2. A total of 65 mutations were found, among which 45 sites were detected in the non-UTR region; 19 were synonymous mutations, and 23 were non-synonymous mutations. Of note, three sites were deleted for sample FAHZU0014. The number of mutations in each sample ranged from 1 to 6. Mutations were mostly distributed on ORF1ab. Most of the samples contained C8782T and T28144C mutations (FAHZU0018 was missing at site 8782 because of insufficient coverage at that site). It is worth noting that FAHZU0002 and FAHZU0019 are derived from the same patient with the same sequence similarity.

**Table 1:** Detail information on the SNP in the 19 sequences.

**Sequence name**	**Clade**	**Missing sites**	**Gaps**	**Mutations**	**Variant type**	**Gene**	**Protein**
FAHZU0002	19B	21	0	C8782T	Synonymous	ORF1a	Ser2839Ser
				G18598A	Missense	ORF1b	Ala1711Thr
				T28144C	Missense	ORF8	Leu84Ser
				C29095T	Synonymous	N	Phe274Phe
				C29586T	Missense	ORF10	Pro10Leu
FAHZU0007	19A	1065	0	C21219T	Synonymous	ORF1ab	Phe6985Phe
FAHZU0008	19A	68	0	T27432C	Synonymous	ORF7a	Ala13Ala
				C28253T	Synonymous	ORF8	Phe120Phe
FAHZU0010	19B	53	0	C8782T	Synonymous	ORF1a	Ser2839Ser
				T28144C	Missense	ORF8	Leu84Ser
FAHZU0011	19A	1195	0	G11083T	Missense	ORF1a	Leu3606Phe
FAHZU0012	19B	40	0	G29742A	Downstream	S	
				C8782T	Synonymous	ORF1a	Ser2839Ser
				C1077T	Missense	ORF1a	Pro271Leu
				G23438T	Missense	S	Ala626Ser
				T28144C	Missense	ORF8	Leu84Ser
				G28878A	Missense	N	Ser202Asn
FAHZU0014	19B	53	3^*^	C8782T	Synonymous	ORF1a	Ser2839Ser
				C19185T	Synonymous	ORF1b	Cys1906Cys
				C16375T	Missense	ORF1b	Pro970Ser
				T28144C	Missense	ORF8	Leu84Ser
FAHZU0017	19A	543	0	C29303T	Missense	N	Pro344Ser
				C15324T	Synonymous	ORF1b	Asn619Asn
FAHZU0018	19B	2406	0	C29095T	Synonymous	N	Phe274Phe
				T28144C	Missense	ORF8	Leu84Ser
				G18598A	Missense	ORF1b	Ala1711Thr
FAHZU0019	19B	24	0	C8782T	Synonymous	ORF1a	Ser2839Ser
				C29095T	Synonymous	N	Phe274Phe
				G18598A	Missense	ORF1b	Ala1711Thr
				T28144C	Missense	ORF8	Leu84Ser
				C29586T	Missense	ORF10	Pro10Leu
FAHZU0020	19A	117	0	C16596T	Synonymous	ORF1b	Tyr1043Tyr
				G26144T	Missense	ORF3a	Gly251Val
FAHZU0021	19A	51	0	C21219T	Synonymous	ORF1b	Phe2584Phe
				G11083T	Missense	ORF1a	Leu3606Phe
FAHZU0022	19A	266	0	C21707T	Missense	S	His49Tyr
				C9711T	Missense	ORF1a	Ser3149Phe
FAHZU0028	19B	1036	0	C1912T	Synonymous	ORF1a	Ser549Ser
				C8782T	Synonymous	ORF1a	Ser2839Ser
				T28144C	Missense	ORF8	Leu84Ser
				C16393T	Missense	ORF1b	Pro976Ser
				C18570T	Synonymous	ORF1b	Leu1701Leu
FAHZU0032	19A	70	0	A21141G	Synonymous	ORF1b	Leu2558Leu
				C28657T	Synonymous	N	Asp128Asp
				G3483T	Missense	ORF1a	Gly1073Val
				G26144T	Missense	ORF3a	Gly251Val
				C27928A	Missense	ORF8	Thr12Asn
FAHZU0033	19B	49	0	A1015G	Synonymous	ORF1a	Glu250Glu
				C8782T	Synonymous	ORF1a	Ser2839Ser
				G17594A	Missense	ORF1b	Ser1376Asn
				A26664G	Missense	M	Ile48Val
				T28144C	Missense	ORF8	Leu84Ser
FAHZU0034	19A	47	0	C6982T	Synonymous	ORF1a	Cys2239Cys
				C19386T	Synonymous	ORF1b	Asp1973Asp
				G22081A	Synonymous	S	Gln173Gln
				T29483G	Missense	N	Ser404Ala
FAHZU0035	19B	33	0	C8782T	Synonymous	ORF1a	Ser2839Ser
				T18603C	Synonymous	ORF1b	His1712His
				C29095T	Synonymous	N	Phe274Phe
				G20683T	Missense	ORF1b	Val2406Phe
				C27925T	Missense	ORF8	Thr11Ile
				T28144C	Missense	ORF8	Leu84Ser
FAHZU0036	19A	1270	0	C486T	Missense	ORF1a	Ser74Leu
				C18512T	Missense	ORF1b	Pro1682Leu
				T18738C	Synonymous	ORF1b	Phe1757Phe

*Mutations are called relative to the reference sequence Wuhan-Hu-1. Unsequenced regions at the 5′ and 3′ end are not shown. Clade names are assigned by the Nextclade*.

* *Three continuous gaps are inserted at position 21991*.

**Figure 1:**
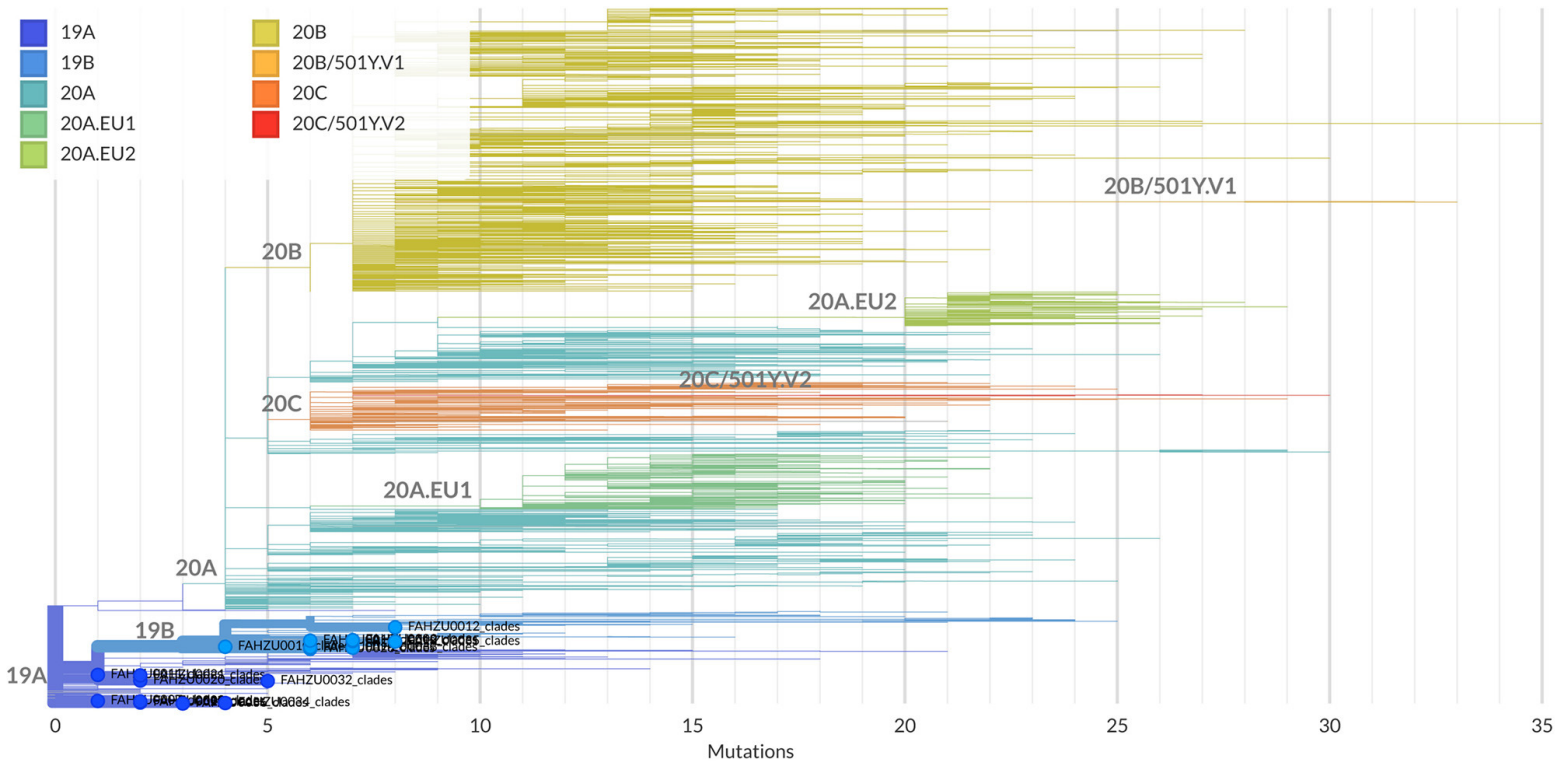
Clade assignment of the 19 Henan sequences analyzed by the Nextclade. Currently, five major clades are defined: 19A and 19B emerged in Wuhan and have dominated the early outbreak; 20A emerged from 19A out of dominated the European outbreak in March and has since spread globally; 20B and 20C are large genetically distinct subclades 20A. The 19 Henan sequences are highlighted and marked with solid circles at the end of their branches.

Clades defined by specific signature mutations are shown in Figure 1. The 19 Henan sequences were located in 19A and19B clades, which emerged in Wuhan and have dominated the early outbreak.

### Demographic, Baseline, and Clinical Characteristics of the Patients

In this study, we only investigated the 18 severe or critically ill patients with COVID-19, for whom sequences of SARS-CoV-2 were successfully assembled with coverage of more than 90% by the aforementioned method. None of these patients were the medical staff. Demographic, baseline, and clinical characteristics of patients are shown in Table 2. Among them, 14 patients (77.78%) were male. Seven (38.89%) patients were exposed to confirmed cases. Four (22.22%) patients traveled to Hubei Province. Fourteen patients (77.78%) were aged 60–89 years, and 4 patients (22.22%) were aged 30–49 years. The minimum age was 30, and the maximum age was 83. Among all 18 patients, 11 (61.11%) had a history of chronic diseases, including cardiovascular and cerebrovascular diseases, cerebrovascular diseases, endocrine system diseases, respiratory system diseases, malignant tumor, chronic kidney disease, chronic liver disease, chronic obstructive pulmonary disease. The most common symptoms were fever and cough that were found in 18 (100.00%) and 13 patients (72.22%), respectively. Eleven patients (61.11%) had shortness of breath. In addition, 1 (5.55%) patient had muscle ache, 2 patients (11.11%) had a headache and mental disorder symptoms, 1 (5.55%) patient had rhinorrhea, and 1 (5.55%) patient had diarrhea. Seventeen patients (94.44%) had more than 1 sign or symptom. All the 18 patients had bilateral pneumonia, according to chest radiograph and CT findings. All patients received antiviral therapy. Seventeen patients (94.44%) were treated with antibiotics. Thirteen (72.22%) received hormone therapy. Fourteen patients (77.78%) received intravenous immunoglobulin therapy. Thirteen patients (72.22%) received mechanical ventilation. Ten patients (55.56%) received ECMO. Eleven (61.11%) received convalescent plasma therapy. Nine (50.00%) received traditional Chinese medicine. Fourteen patients (77.78%) were negative for nucleic acid tests and continued to receive treatment for other underlying diseases, and 4 patients (22.22%) died.

**Table 2:** Demographic, baseline, and clinical characteristics of the 18 patients with COVID-19 in Henan Province.

**Variables**	**Patients (*N* = 18)**
**Age, years**	
Mean ± SD	65.11 ± 16.78
Age group, n (%)	
<29 years	0 (0.00)
30-39 years	2 (11.11)
40-49 years	2 (11.11)
50-59 years	0 (0.00)
60-69 years	5 (27.78)
70-79 years	6 (33.33)
80-89 years	3 (16.67)
≥90 years	0 (0.00)
**Sex, n (%)**	
Female	4 (22.22)
Male	14 (77.78)
**Clinical classification, n (%)**	
Mild type	0 (0.00)
Moderate type	0 (0.00)
Severe type	6 (33.33)
Critically ill type	12 (66.67)
**Exposure to confirmed cases, n (%)**	
Yes	7 (38.89)
No	11 (61.11)
**Travel to Hubei**	
Yes	4 (22.22)
No	14 (77.73)
Comorbidities, n (%)	11 (61.11)
Cardiovascular and cerebrovascular diseases, cerebrovascular diseases	5 (27.78)
Endocrine system diseases	4 (22.22)
Respiratory system diseases	1 (5.55)
Malignant tumor	1 (5.55)
Nervous system diseases	0 (0.00)
Chronic kidney disease	1 (5.55)
Chronic liver disease	1 (5.55)
Chronic obstructive pulmonary disease	1 (5.55)
More than 1 comorbidity	4 (22.22)
**Signs and symptoms at admission, n (%)**	
Fever	18 (100)
Cough	13 (72.22)
Shortness of breath	11 (61.11)
Muscle ache	1 (5.55)
Headache and mental disorder symptoms	2 (11.11)
Sore throat	2 (11.11)
Rhinorrhea	1 (5.55)
Chest pain	0 (0.00)
Diarrhea	1 (5.55)
Nausea and vomiting	0 (0.00)
More than 1 sign or symptom	17 (94.44)
**Chest radiograph and CT findings, n (%)**	
Bilateral pneumonia	18 (100.00)
Unilateral pneumonia	0 (0.00)
No abnormal density shadow	0 (0.00)
**Treatment, n (%)**	
Antibiotic treatment	17 (94.44)
Antiviral treatment	18 (100.00)
Hormone therapy	13 (72.22)
Intravenous immunoglobulin therapy	14 (77.78)
Mechanical ventilation	13 (72.22)
ECMO	10 (55.56)
Convalescent plasma therapy	11 (61.11)
Traditional Chinese medicine	9 (50.00)
**Clinical outcome, n (%)**	
Negative and discharged	14 (77.78)
Died	4 (22.22)

*CT, computed tomography; ECMO, extracorporeal membrane oxygenation; SD, standard deviation*.

### Sequences Distribution

**Figure 2:**
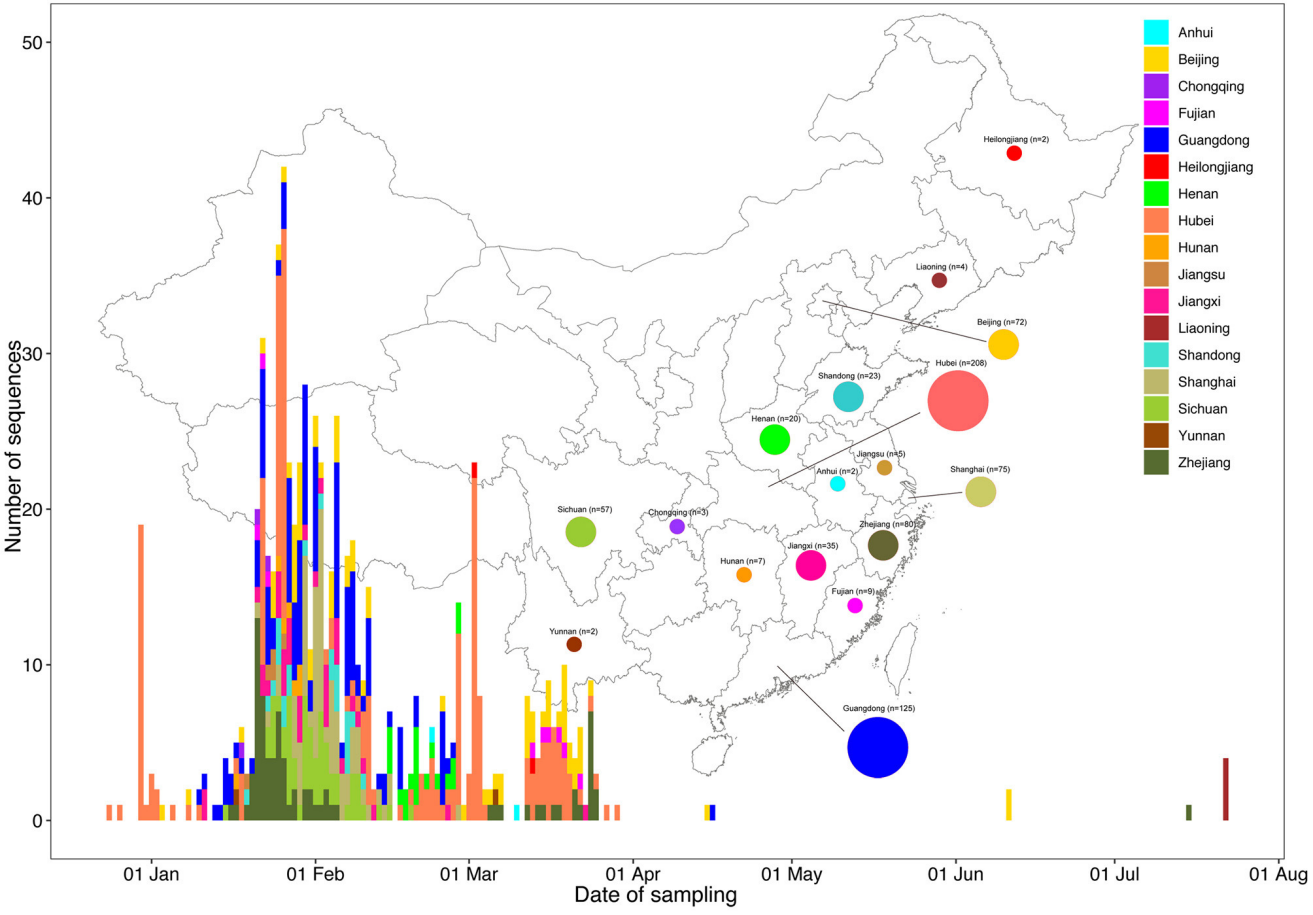
Time series and geographic distribution of the 729 SARS-CoV-2 genomes from Mainland China by sampling date. The geographic distribution of the 729 SARS-CoV-2 genomes from Mainland China in the present study is shown at the provincial level. Colors indicate different sampling provinces from Mainland China.

As of October 23, 2020, 710 complete genomes of SARS-CoV-2 detected in mainland China were available from GISAID (http://gisaid.org/) (Supplementary Table 1). The sequences classified by sampling provinces through sampling dates are shown in Figure 2. Together with 19 sequences obtained from Henan Province in this study, our dataset finally (“Dataset_729”) included 729 genome sequences of SARS-CoV-2 sampled from 17 provinces in mainland China (Figure 2. Anhui, *n* = 2; Beijing, *n* = 72; Chongqing, *n* = 3; Fujian, *n* = 9; Guangdong, *n* = 125; Heilongjiang, *n* = 2; Henan, *n* = 20; Hubei, *n* = 208; Hunan, *n* = 7; Jiangsu, *n* = 5; Jiangxi, *n* = 35; Liaoning, *n* = 4; Shandong, *n* = 23; Shanghai, *n* = 75; Sichuan, *n* = 57; Yunnan, *n* = 2; and Zhejiang, *n* = 80) with sampling dates between 24 December 2019 and 22 July 2020. The sequences were primarily from Hubei (208/729, 28.53%) and Guangdong (125/729, 17.15%). It is worth mentioning that the sequence distribution with reference to sampling time and province was very uneven; there were few sequences after April 1, 2020 due to the successful control of the epidemic in China.

### Tree-Like Signals and Phylogenetic Analyses

**Figure 3:**
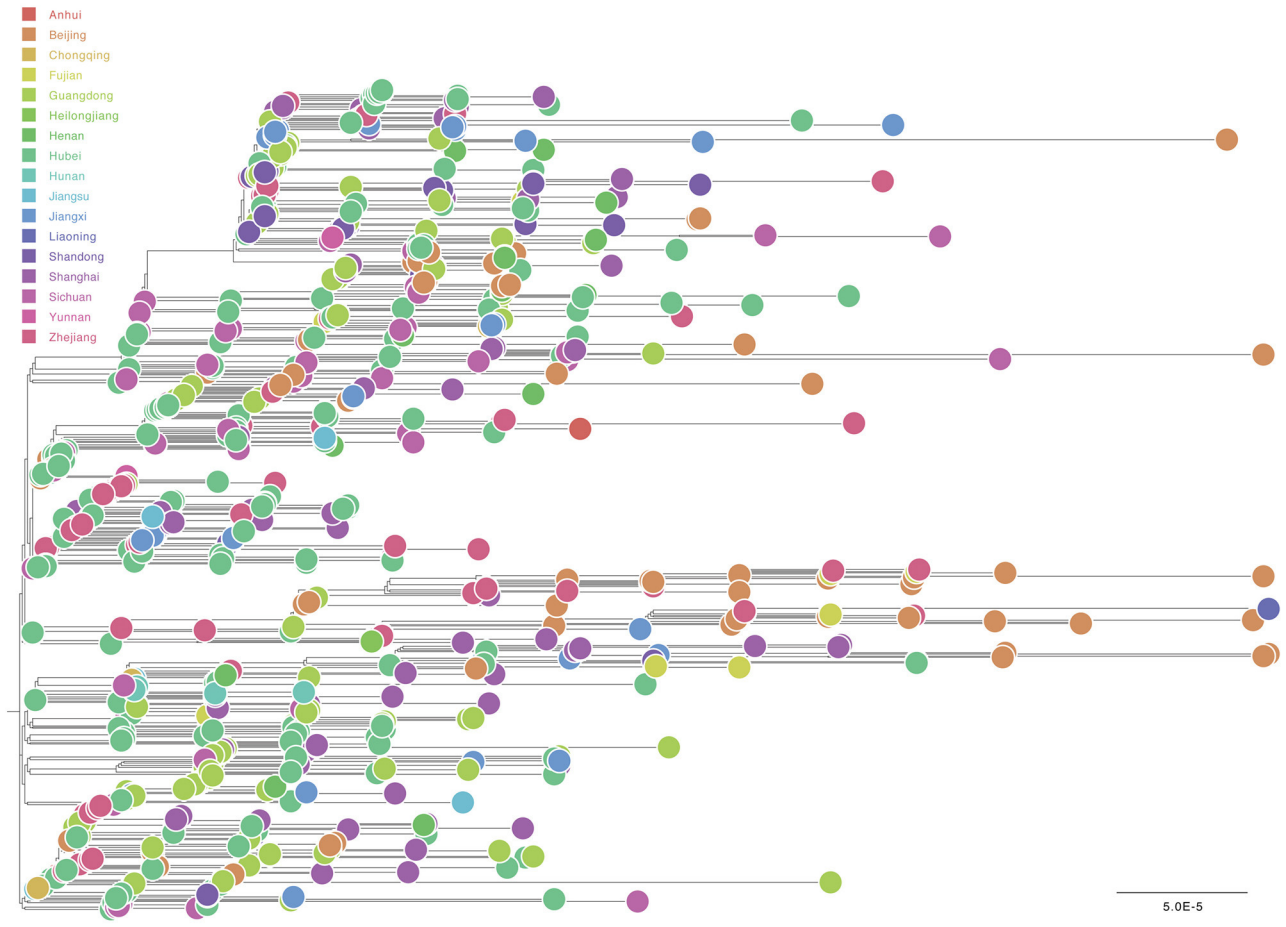
Estimated maximum-likelihood phylogenetic tree of SARS-CoV-2 from Mainland China. Maximum-likelihood phylogenetic tree of SARS-CoV-2 for “dataset_729” from Mainland China is shown. Tree is midpoint rooted. Colors indicate different sampling provinces from Mainland China. The scale bar at the bottom indicates 0.00005 nucleotide substitutions per site.

For “dataset_729,” a GTR+Γ+I nucleotide substitution model was the best-fit model based on the three substitution schemes (i.e., 24 candidate models) according to the AIC, AICc, BIC, and DT methods, and was thus used in subsequent likelihood-mapping and phylogenetic analyses. The PHI test of “dataset_729” showed no statistically significant evidence of recombination (*p* = 1.0). In addition, no evidence of recombination was found for “dataset_729” using RDP v4.100 (Martin et al., 2015). Our likelihood-mapping analysis revealed that the quartets from “dataset_729” were primarily distributed in the center (56.7%) rather than the corners (43%) or sides (0.3%) of the triangle, indicating a strong star-like topology signal and suggesting the possible rapid early spread of SARS-CoV-2 (Supplementary Figure 3A), which is in accordance with previous studies (Li et al., 2020b; Nie et al., 2020). The split network generated for “dataset_729” using the NeighborNet method revealed the existence of polytomies and thus was highly unresolved. This indicated that the phylogenetic relationship of our dataset was probably best represented by a star-like phylogenetic tree rather than a strictly bifurcating tree (Supplementary Figure 3B) that may be due to exponential epidemic spread, which was in accordance with the likelihood-mapping results. ML phylogenetic analysis of “dataset_729” also showed star-like topology (Figure 3), indicating the introduction of a new virus to an immunologically naive population, which was in accordance with the likelihood-mapping and split network results.

Notably, most genomic sequences of SARS-CoV-2 sampled from Henan Province were clustered with genomic sequences sampled from three other Chinese provinces: Guangdong, Sichuan, and Hubei (Supplementary Figure 4), thus indicating that SARS-CoV-2 had already spread from Hubei Province to other Chinese provinces, and also revealing that multiple independent introductions of SARS-CoV-2 from other Chinese provinces into Henan Province had already occurred, although phylogenetic analyses were limited due to the low genetic variation of the virus.

**Figure 4:**
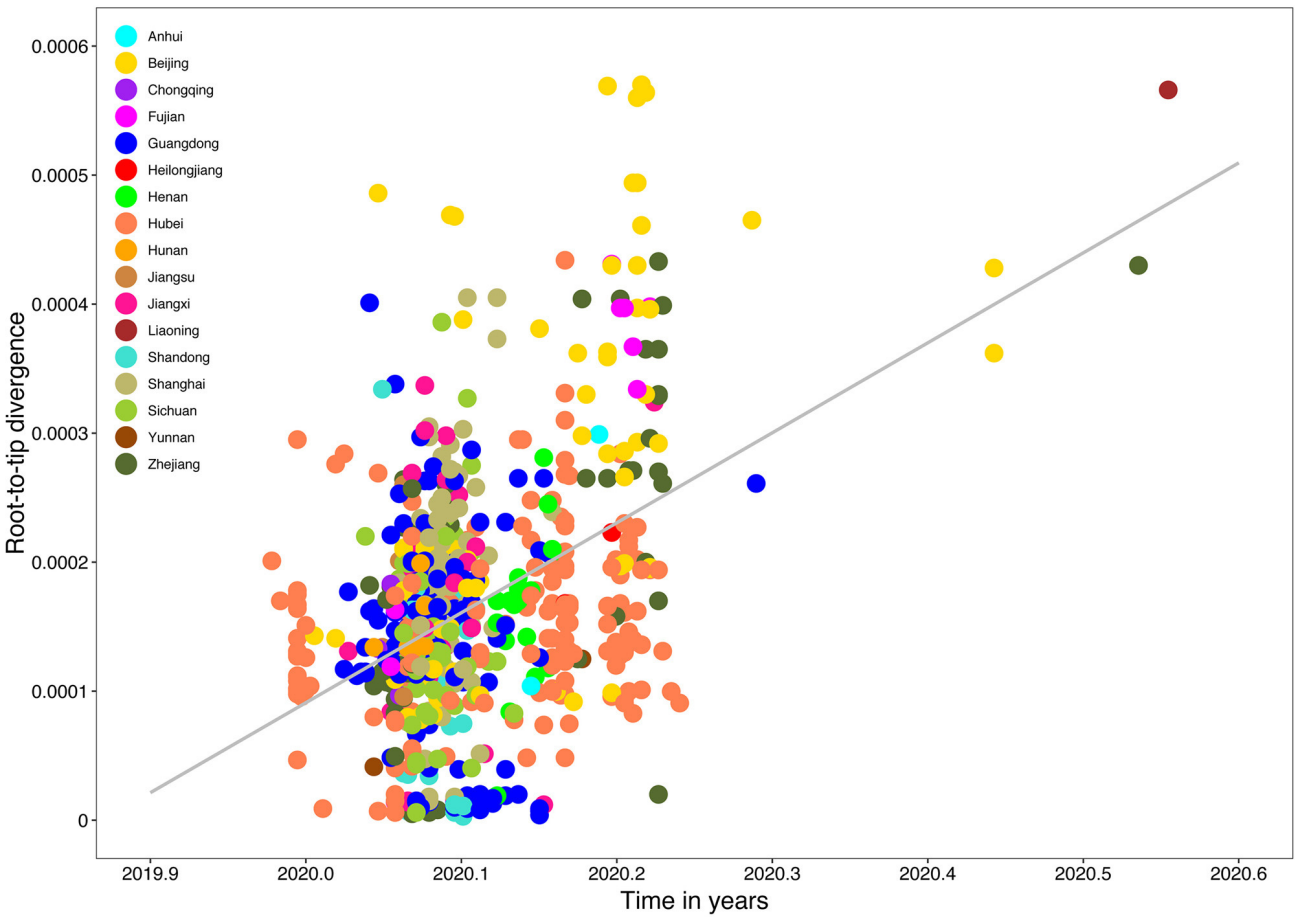
Root-to-tip genetic divergence plot of SARS-CoV-2 from Mainland China. Root-to-tip genetic divergence for “dataset_729” from Mainland China in the Maximum likelihood tree (as shown in Figure 3) plotted against sampling date is shown. Colors indicate different sampling provinces from Mainland China. Gray color indicates linear regression line.

**Figure 5:**
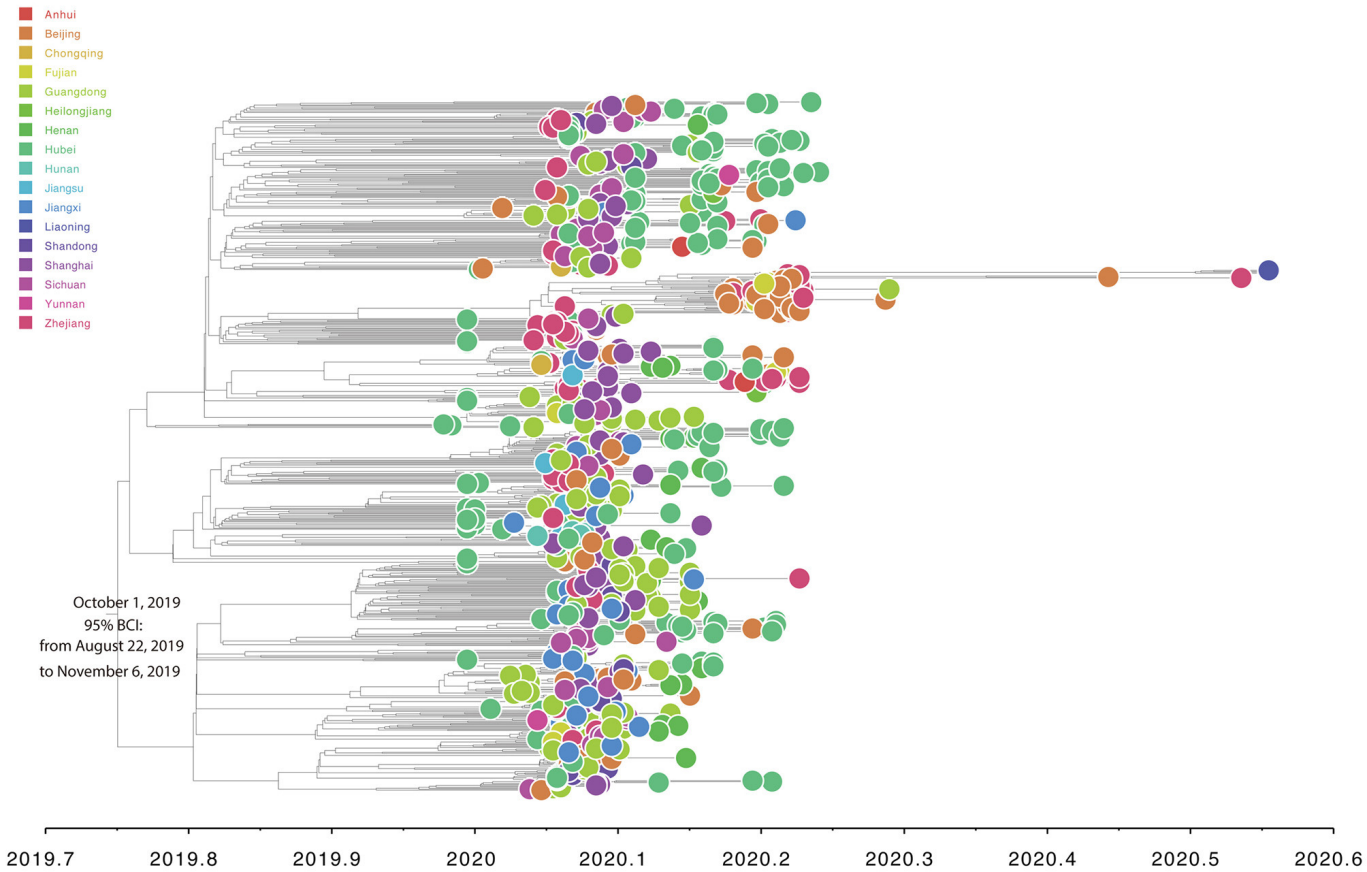
Estimated Bayesian time-scaled maximum-clade-credibility phylogenetic tree of SARS-CoV-2 from Mainland China. Circle at the tip is colored according to sampling provinces from Mainland China. Note that the axis of abscissas is scaled by decimal date.

Root-to-tip linear regression analyses between genetic divergence and sampling date using the best-fitting root, which minimizes the mean of the squares of the residuals, showed that “dataset_729” had a positive temporal signal (*R*^2^ = 0.22; correlation coefficient = 0.47), thus suggesting a clocklike pattern of molecular evolution (Figure 4). The evolutionary rate and TMRCA date estimates of SARS-CoV-2 for “dataset_729” were 6.9735 × 10^−4^ substitutions per site per year and November 14, 2019, respectively. Based on Bayesian time-scaled phylogenetic analysis using the tip-dating method, the estimated TMRCA date and evolutionary rate estimates of SARS-CoV-2 for “dataset_729” under a strict molecular clock along with an exponential growth tree prior model with growth rate parameterization using the tip-dating method were October 1, 2019 [95% Bayesian credible interval (BCI): August 22, 2019 to November 6, 2019] and 9.25 × 10^−4^ substitutions per site per year (95% BCI: 6.75 × 10^−4^ to 1.28 × 10^−3^), respectively. Still, these data were not consistent with the root-to-tip regression results using TempEst v1.5 (Rambaut et al., 2016). However, this posterior evolutionary rate is consistent with previous analyses (Nie et al., 2020; Geidelberg et al., 2021). The estimated TMRCA of the SARS-CoV-2 for “dataset_729” using Bayesian time-scaled phylogenetic analysis were around two months earlier than the result of the previous study (Lu et al., 2020), which may be due to the different datasets and Bayesian time-scaled phylogenetic models used. The estimated growth rate for “dataset_729” under the exponential growth tree prior model was 7.4 per year (95% BCI: 5.1–9.6). The estimates of the MCC phylogenetic relationships among the SARS-CoV-2 genomes for “dataset_729” from the Bayesian coalescent framework using the tip-dating method, as well as the exponential coalescent tree prior with growth rate parameterization and a strict molecular clock model, are displayed in Figure 5. As shown, “dataset_729” exhibited more genetic diversity than previous datasets (Li et al., 2020a,b,c; Nie et al., 2020).

### Discussion

In the present study, no mutations were found in the viral sequences from the same patient collected at two time points with a short interval. This might be due to the virus has a very low probability of mutating *in vivo* over a relatively limited time. However, it has been shown that the virus mutates in chronically infected patients (Kemp et al., 2021). For a better understanding of the impact of the minority viral population on SARS-CoV-2 evolution and transmission, more studies are needed to uncover the in-host variability of the virus during the course of the infection.

To investigate the epidemic spread of SARS-CoV-2 in Mainland China, we performed comprehensive evolutionary analyses of 729 genomes from “dataset_729.” Our analyses of the genomic epidemiology of SARS-CoV-2 in Mainland China indicated that most infections from Henan Province resulted from virus importation from three other Chinese provinces, Guangdong, Zhejiang, and Hubei (Figure 3 and Supplementary Figure 4). Also, multiple independent introductions of SARS-CoV-2 from other Chinese provinces into Henan Province were detected. Previous study have also reported multiple independent introductions of SARS-CoV-2 into Guangdong Province (Lu et al., 2020), and Weifang city of Shandong Province; yet, the epidemic spread of SARS-CoV-2 in Henan Province, Guangdong Province and Shandong Province was limited in size and duration. However, all these studies indicated that China has already contained the COVID-19 epidemic in a very short time period. The large-scale surveillance and intervention measures implemented in Henan Province effectively interrupted local transmission and ultimately contained the epidemic and limited eventual dissemination to other regions. Therefore, pathogen sequences have a large potential to inform epidemic surveillance and intervention efforts. It is worth noting that analyses of the phylogenetic structure should be carefully interpreted, as the number of mutations used for the phylogenetic structure was small. It is worth mentioning that the present study was based on a low and variable sampling of COVID-19 cases among different provinces in Mainland China and that COVID-19 cases from other provinces were still not sampled or sequenced or available at GISAID. Nationally, the obtained full-genome sequences of SARS-CoV-2 viruses represent only a tiny fraction of China's number of actual infections. Therefore, it is not suitable to draw conclusions on unknown early genetic diversity and geographic transmission routes in China based on such a small and under-sampled data set. Therefore, more genome sequences of SARS-CoV-2 are needed for a better understanding of the spread of the epidemic patterns of SARS-CoV-2 in China.

In conclusion, this study characterized the epidemic spread patterns of SARS-CoV-2 in Mainland China (including 17 provinces) based on genome data obtained from patients with COVID-19 between December 24, 2019 and July22, 2020. The genomes of SARS-CoV-2 obtained from Henan Province are important for regional and national efforts and other countries to understand how this virus is evolving and spreading around the world. Our results further the understanding of the molecular epidemiology and genetic diversity of SARS-CoV-2 over time in Mainland China. Our results also emphasize the importance of combining the SARS-CoV-2 genomes with all available epidemiological information for all phylogenetic analyses to gain insights into the roles of various surveillance and intervention measures for containing the spread of SARS-CoV-2 in China. Understanding antigenic evolution of SARS-CoV-2, especially in the viral spike, and more especially in the receptor-binding domain in real-time, is increasingly important for guiding prevention efforts and updating SARS-CoV-2 vaccines and treatments. More importantly, the public should also continue to take action in their area to reduce SARS-CoV-2 transmission.

## Data Availability Statement

The datasets presented in this study can be found in online repositories. The names of the repository/repositories and accession number(s) can be found in the article/Supplementary Material.

## Ethics Statement

The studies involving human participants were reviewed and approved by the ethics committee of the First Affiliated Hospital of Zhengzhou University. The ethics committee waived the requirement of written informed consent for participation.

## Author Contributions

Q-LZ and NS conceived and designed the study. NS and G-LC analyzed the data and wrote this manuscript. Q-LZ interpreted the data and provided critical comments. All authors reviewed and approved the final manuscript.

## Conflict of Interest

The authors declare that the research was conducted in the absence of any commercial or financial relationships that could be construed as a potential conflict of interest.

## Supplementary Material

Supplementary Figure 1 The mutation site plot for the 19 samples with a coverage >90% obtained from the present study. Orange color presents mutation sites in each SARS-CoV-2 genome sequence.

Supplementary Figure 2 Sequences view with the mapping information of their mutations, missing data, and gaps. Mutations are called relative to the reference sequence Wuhan-Hu-1. Ns (missing) displays a number of N characters (missing data) in the sequence. Each row displays a schema of the corresponding sequence by highlighting the differences to the reference Wuhan-Hu-1 along the genome. Positions are 1-based. Line markers on sequence views represent mutations colored by the resulting nucleotide, as shown at the top right of the figure. Unsequenced regions at the 5′ and 3′ end are indicated as light gray shading. The genome annotation view below the table displays the mapping between positions in the sequence, genes, and clade-defining mutations. It should be notedthat sometimes mutations are so close to each other that they overlap. Clade names are assigned by the Nextclade. Detailed information on the SNP is shown in Supplementary Table 1.

Supplementary Figure 3 Likelihood-mapping and split network analyses of SARS-CoV-2 from Mainland China. Likelihood-mapping **(A)** and split network **(B)** analyses of SARS-CoV-2 for “dataset_729” from Mainland China with sampling dates between December 24, 2019 and July 22, 2020 are shown.

Supplementary Figure 4 Detailed maximum-likelihood phylogenetic tree of SARS-CoV-2 from Mainland China. This tree shows the detailed information of Figure 3.

Supplementary Table 1 Detail information on the 710 SARS-CoV-2 genomic sequences data set downloaded from GISAID.

Supplementary Table 2 Assembly information of the 28 nucleic acid samples obtained from the present study.
