# Supplementary figures and images for: Genomic Epidemiology of SARS-CoV-2 From Mainland China With Newly Obtained Genomes From Henan Province
Source: Front Microbiol. 2021 May 20;12:673855. doi: 10.3389/fmicb.2021.673855 (PMC8172800; doi:10.3389/fmicb.2021.673855)

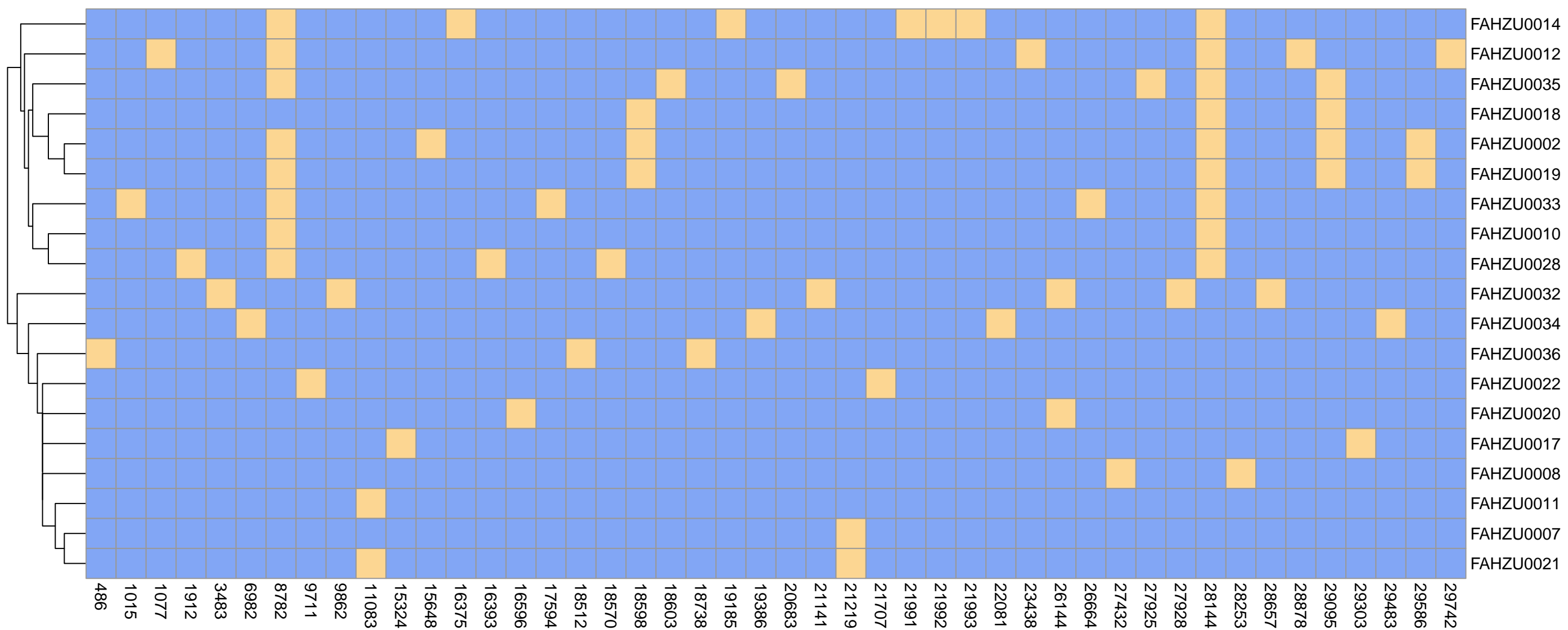

Supplement: Supplementary Figure 1 — The mutation site plot for the 19 samples with a coverage >90% obtained from the present study. Orange color presents mutation sites in each SARS-CoV-2 genome sequence. [file Data_Sheet_1.PDF]

A

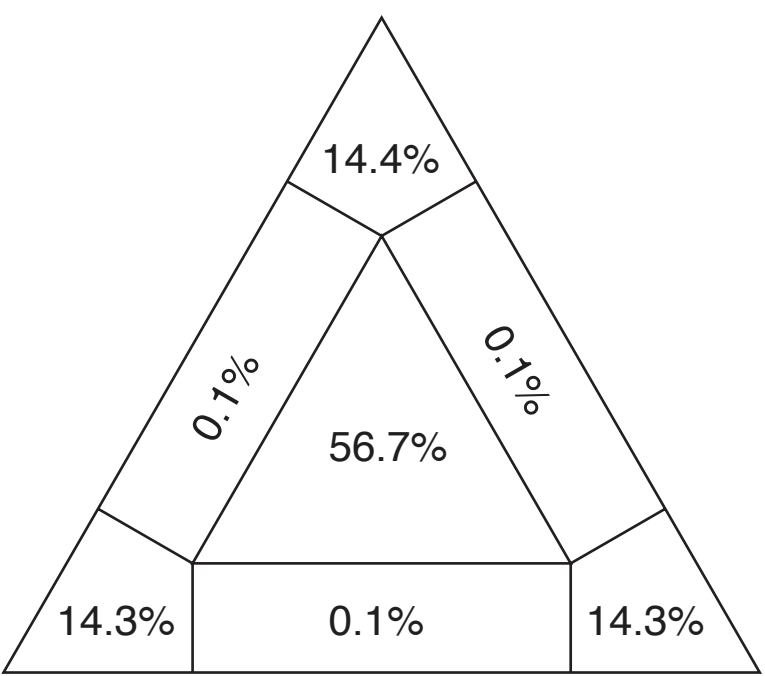

B

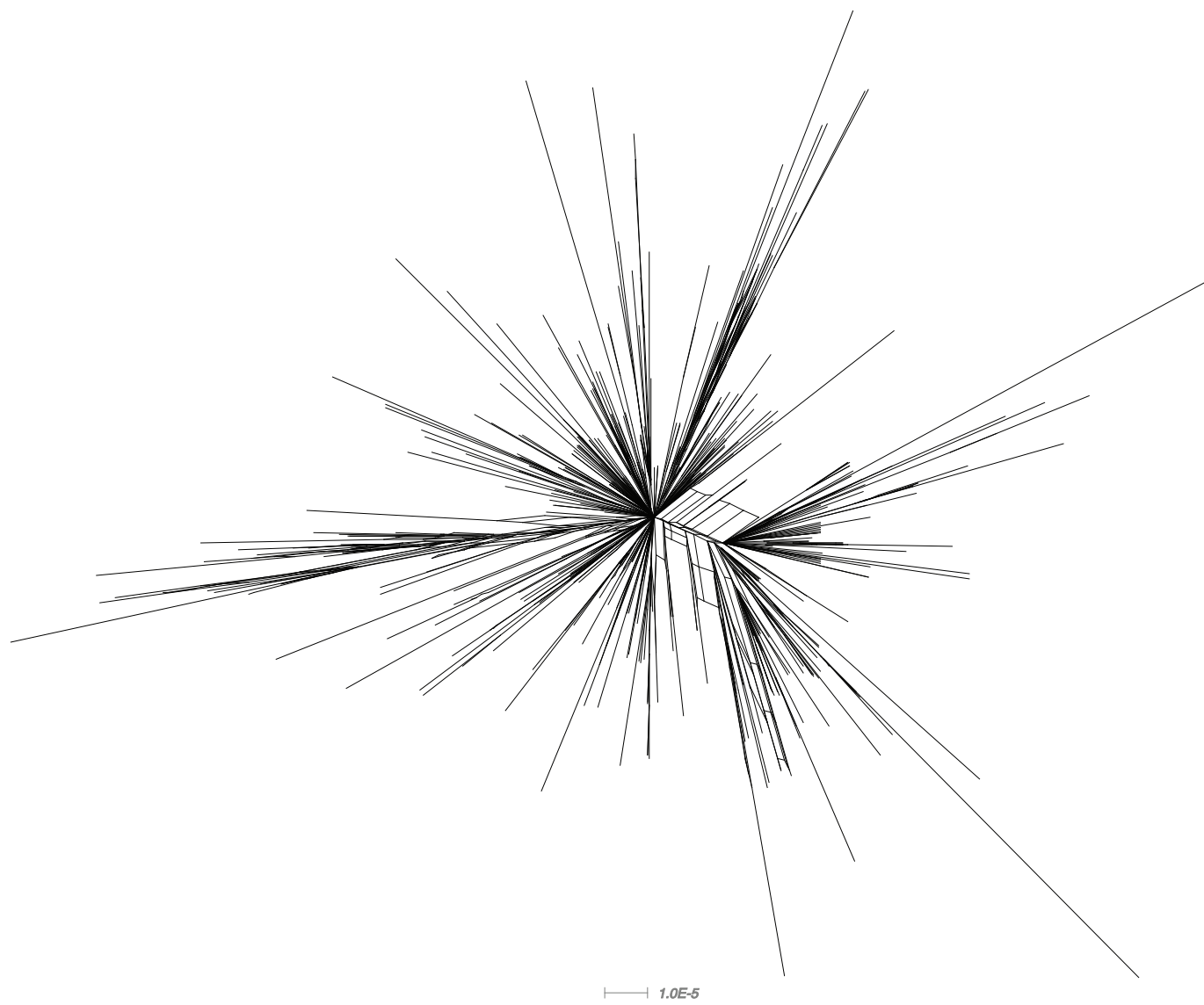

Supplement: Supplementary Figure 2 — Sequences view with the mapping information of their mutations, missing data, and gaps. Mutations are called relative to the reference sequence Wuhan-Hu-1. Ns (missing) displays a number of N characters (missing data) in the sequence. Each row displays a schema of the corresponding sequence by highlighting the differences to the reference Wuhan-Hu-1 along the genome. Positions are 1-based. Line markers on sequence views represent mutations colored by the resulting nucleotide, as shown at the top right of the figure. Unsequenced regions at the 5′ and 3′ end are indicated as light gray shading. The genome annotation view below the table displays the mapping between positions in the sequence, genes, and clade-defining mutations. It should be notedthat sometimes mutations are so close to each other that they overlap. Clade names are assigned by the Nextclade. Detailed information on the SNP is shown in Supplementary Table 1. [file Data_Sheet_2.PDF]

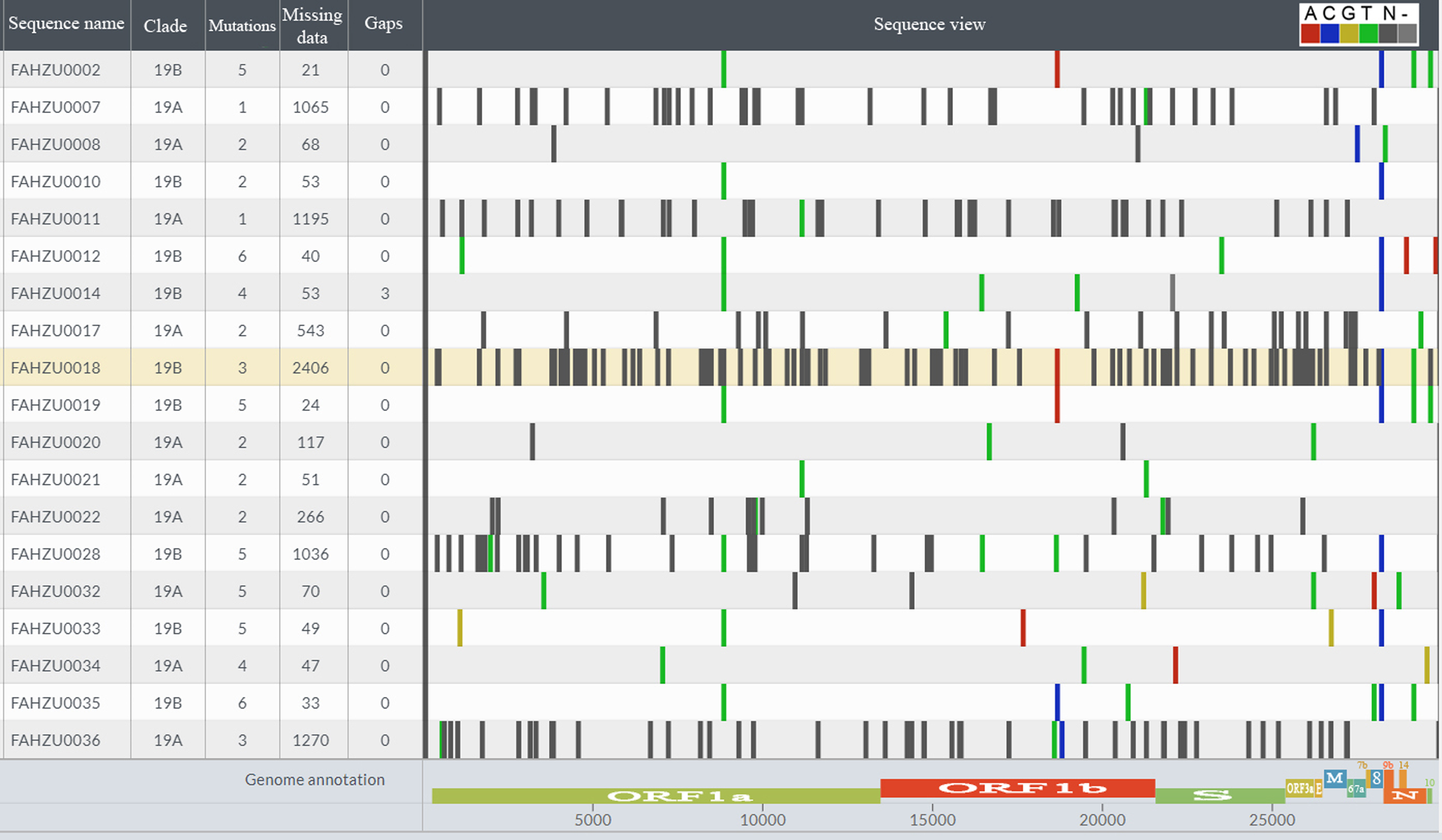

Supplement: Supplementary Figure 4 — Detailed maximum-likelihood phylogenetic tree of SARS-CoV-2 from Mainland China. This tree shows the detailed information of Figure 3. [file Image_1.JPEG]
