# Supplementary material for: Genomic Epidemiology of SARS-CoV-2 From Mainland China With Newly Obtained Genomes From Henan Province
Source: Front Microbiol. 2021 May 20;12:673855. doi: 10.3389/fmicb.2021.673855 (PMC8172800; doi:10.3389/fmicb.2021.673855)
Supplement: Supplementary Table 2 — Assembly information of the 28 nucleic acid samples obtained from the present study. [file Data_Sheet_5.PDF]

Table S2. Assembly information of the 28 nucleic acid samples obtained from the present study

ID	Sequencing volume(G)	All reads (pairs)	Reads of SRAS-nCoV-2 (pairs)	NSPM	Coverage	Depth	assembled length
FAHZU0001	101.0455	5.05E+08	1094	2.165361	81.44	2.48626	19406
FAHZU0002	135.0099	6.75E+08	8713187	12907.48	99.98	3299.94	29870
FAHZU0003	172.7221	8.64E+08	670.5	0.776391	62.9	1.33763	15965
FAHZU0004	149.5766	7.48E+08	12672.5	16.9445	75.07	2.06417	20079
FAHZU0005	206.9569	1.03E+09	1371	1.324914	86.24	3.0288	23939
FAHZU0007	129.5396	6.48E+08	14998.5	23.15662	98.4	6.73172	28833
FAHZU0008	41.96773	2.1E+08	157336	749.7952	99.89	20.672	29823
FAHZU0010	97.73775	4.89E+08	611986	1252.302	99.89	40.6835	29838
FAHZU0011	175.7749	8.79E+08	1418	1.613427	97.95	6.58416	28701
FAHZU0012	124.8538	6.24E+08	555927	890.5248	99.93	111.795	29851
FAHZU0014	152.1937	7.61E+08	9746	12.80737	99.89	47.9626	29838
FAHZU0016	55.81729	2.79E+08	22.5	0.08062	8.57	0.128467	1583
FAHZU0017	196.722	9.84E+08	1639.5	1.666819	99.3	8.52618	29349
FAHZU0018	77.49249	3.87E+08	1030	2.658322	96.15	4.12897	27488
FAHZU0019	185.6759	9.28E+08	4479760	4825.355	99.98	6148.07	29867
FAHZU0020	95.86879	4.79E+08	6113	12.75285	99.75	10.937	29774
FAHZU0021	129.8728	6.49E+08	25075	38.61471	99.9	34.2307	29840
FAHZU0022	108.4731	5.42E+08	2343	4.319966	99.74	15.4594	29628
FAHZU0023	216.0329	1.08E+09	796	0.736925	72.49	1.84985	19232
FAHZU0024	96.84407	4.84E+08	514.5	1.062533	79.3	2.41163	21666
FAHZU0027	268.4162	1.34E+09	153.5	0.114375	50.09	0.87916	13153
FAHZU0028	177.8519	8.89E+08	1797.5	2.021344	97.81	7.58178	28857
FAHZU0029	140.4712	7.02E+08	914	1.301334	88.13	2.99281	24833
FAHZU0032	169.0629	8.45E+08	10549	12.47938	99.89	13.3803	29821
FAHZU0033	111.8791	5.59E+08	38989	69.69843	99.9	25.9003	29842
FAHZU0034	104.3131	5.22E+08	39590.5	75.90703	99.91	59.633	29844
FAHZU0035	308.0729	1.54E+09	72246	46.90188	99.96	295.871	29858
FAHZU0036	233.6775	1.17E+09	1005	0.86016	97.8	6.376	28628
