# Supplementary material for: Genomic Epidemiology of SARS-CoV-2 From Mainland China With Newly Obtained Genomes From Henan Province
Source: Front Microbiol. 2021 May 20;12:673855. doi: 10.3389/fmicb.2021.673855 (PMC8172800; doi:10.3389/fmicb.2021.673855)
Supplement: Supplementary Figure 3 — Likelihood-mapping and split network analyses of SARS-CoV-2 from Mainland China. Likelihood-mapping (A) and split network (B) analyses of SARS-CoV-2 for “dataset_729” from Mainland China with sampling dates between December 24, 2019 and July 22, 2020 are shown. [file Data_Sheet_3.PDF]

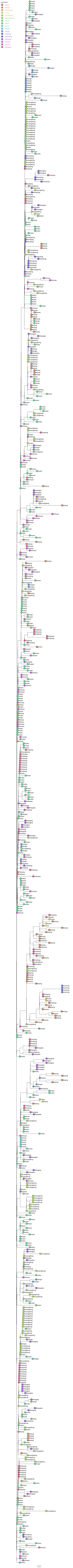
province
Anhui
Beijing
Chongqing
Fujian
Guangdong
Heilongjiang
Henan
Hubei
Hunan
Jiangsu
Jiangxi
Liaoning
Shandong
Shanghai
Sichuan
Yunnan
Zhejiang
